# Polymeric Delivery System for mRNA Therapeutics: Design Principles and Recent Advances

**DOI:** 10.3390/genes17060646

**Published:** 2026-05-31

**Authors:** Sidi Bao, Irene Rose Reuben, Josie Ward, Wenxin Wang, Xianqing Wang

**Affiliations:** Charles Institute of Dermatology, School of Medicine, University College Dublin, D04 V1W8 Dublin, Ireland; sidi.bao@ucdconnect.ie (S.B.); irene.reuben@ucdconnect.ie (I.R.R.); josie.ward@ucd.ie (J.W.); wenxin.wang@ucd.ie (W.W.)

**Keywords:** mRNA therapeutics, polymeric vectors, poly(beta-amino ester), polyethyleneimine, CART, dendrimer, hybrid nanoparticles, endosomal escape, targeted delivery

## Abstract

Messenger RNA (mRNA) therapeutics are redefining treatment approaches in vaccines, cancer immunotherapy, protein replacement, and gene editing. Lipid nanoparticles have enabled early clinical successes, but they can be limited by liver-dominant biodistribution, long-term storage stability, and systemic tolerability. Polymeric delivery systems offer a versatile alternative, with tunable physicochemical properties enabling precise control over mRNA complexation, protection, release, and targeting. This review examines recent progress across polyethyleneimine derivatives, poly(β-amino ester)s, poly(amino acid)s, polyesters, dendrimers, charge-altering releasable transporters, and lipid-polymer hybrids. We highlight strategies such as structural modification, stimuli-responsive designs, and high-throughput polymer screening that enhance stability, reduce cytotoxicity, and enable organ- or cell-specific delivery. Addressing challenges in immunogenicity, biodistribution, and manufacturing scalability will be pivotal to translating these innovations into safe and effective mRNA therapeutics.

## 1. Introduction

In recent years, significant attention has been paid to mRNA-based therapy for its potential in next-generation applications, including cancer vaccines, gene-editing therapies, and protein-replacement therapies. [1]. Messenger RNA (mRNA) differs fundamentally from DNA in that it operates transiently in the cytoplasm, circumventing the risk of genomic integration and facilitating rapid and controlled protein expression in therapeutic applications. The risk of long-term genetic alterations is thus minimized, and precise temporal regulation of therapeutic effects can be carried out. However, its ephemeral activity means that successful delivery of mRNA remains a challenge, due to various factors including inherent instability, susceptibility to degradation by ribonucleases (RNases), inefficient cellular uptake, and intracellular barriers such as endosomal entrapment. To address these challenges, delivery vectors must be strategically designed to optimize successful cellular uptake and expression of mRNA [2].

Nonviral vectors are increasing focus in mRNA delivery research. Although viral vectors can achieve high delivery efficiency, their potential for genome integration and pronounced immunogenicity narrows their therapeutic applicability [3]. In contrast, nonviral delivery systems mitigate these risks while providing superior design flexibility, allowing precise control over pharmacokinetics and release kinetics for diverse therapeutic applications [4]. Amongst these, lipid nanoparticles (LNPs) have been extensively studied in preclinical and clinical settings and have contributed to successful clinical translations, such as integration into approved COVID-19 vaccines. LNPs benefit from the ability to efficiently encapsulate mRNA, providing protection against degradation and enabling cellular uptake. Despite these advantages, conventional LNPs tend to accumulate in the liver due to nonspecific biodistribution, limiting their effectiveness for extrahepatic targets [5]. Further challenges with long-term storage and stability constrain their broader therapeutic application. On that front, polymer-based mRNA carriers have since emerged as promising alternatives suitable for advanced mRNA-based therapies, offering structural versatility, as well as unique pharmacokinetic profiles [6].

Polymeric delivery systems represent a scientifically important and timely area in mRNA therapeutics because they offer a chemically versatile platform to address several key delivery barriers, including mRNA condensation, nuclease protection, cellular uptake, endosomal escape, intracellular release, biodegradability, and tissue-selective delivery. Although lipid nanoparticles have achieved the most advanced clinical translation, polymeric vectors provide distinct opportunities for rational molecular design through the precise tuning of molecular weight, charge density, hydrophobicity, topology, degradability, and functional modification. These features make polymeric systems highly relevant not only as alternative carriers to lipid-based formulations, but also as independent materials platforms for understanding structure–function relationships in mRNA delivery. In this review, we explore recent advances in polymeric mRNA delivery systems, consisting of diverse classes of polymers: polyethyleneimine (PEI), poly(β-amino ester)s (PBAEs), poly(amino acid)s, biodegradable polyesters, dendrimers, charge-altering releasable transporters (CARTs), and lipid/polymer hybrid systems. These polymeric systems have been widely investigated for their capacity to condense mRNA into stable nanoparticle complexes and to facilitate its release into the cytoplasm, thereby improving therapeutic outcomes [6]. There are, however, distinct advantages and challenges associated with each class—for instance, PEI, while known for its efficient nucleic acid condensation, raises concerns regarding cytotoxicity due to its high charge density, which has spurred interest in biodegradable alternatives such as PBAEs and poly(amino acid)s [7]. Similarly, dendrimers and CARTs offer modifiable architectures and release profiles, while lipid-polymer hybrids combine the robustness of polymers with the membrane fusion properties of lipids [1]. By addressing current challenges and emerging opportunities within this field, this review summarizes advanced polymeric systems for mRNA delivery developed in recent years, as well as prospects in therapeutic applications.

## 2. Overview of mRNA Delivery

**Figure 1:**
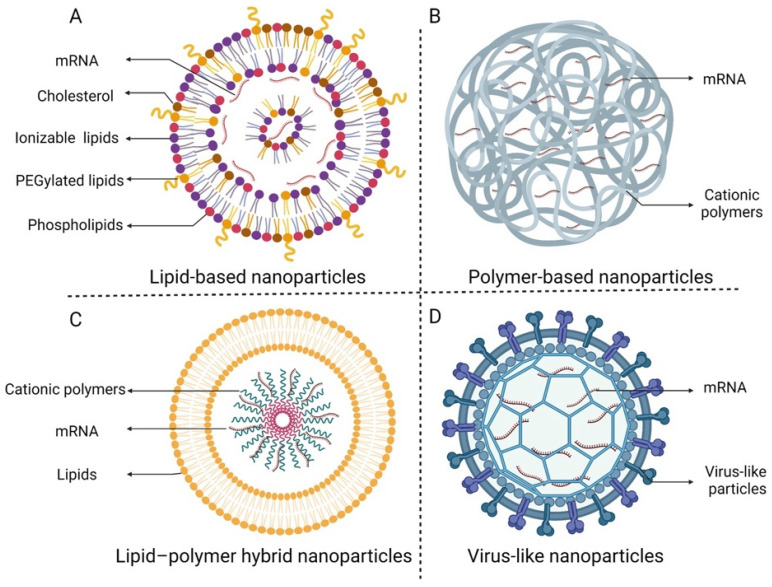
Architectures of nonviral mRNA delivery systems. This figure illustrates four primary delivery platforms: (**A**) lipid nanoparticles (LNPs), which utilize an ionizable lipid core to capture mRNA via electrostatic forces within a shell of cholesterol, phospholipids, and PEGylated lipids; (**B**) polymer-based nanoparticles, which offer a simpler alternative typically consisting of cationic polymers; and (**C**) lipid–polymer hybrids, which integrate these approaches by shielding a polymer-encapsulated mRNA core with a phospholipid outer layer. Lastly, (**D**) virus-like particles (VLPs) leverage viral proteins to create biomimetic structures that safeguard mRNA from degradation while exploiting natural pathways to facilitate efficient cellular entry and cargo release. Reprinted with permission from ref. [4]. Copyright 2024, Elsevier.

The success of mRNA therapeutics relies on delivery systems that overcome the inherent challenges of mRNA instability, inefficient cellular uptake, and immunogenicity (Figure 1) [8,9]. LNPs, exemplified by their pivotal role in COVID-19 vaccines, remain the gold standard [10]. The synergy of components is largely the reason for LNP vectors’ high encapsulation efficiencies and robust transfection across diverse biological contexts. Ionizable lipids remain neutral at physiological pH, thereby reducing systemic charge-based toxicity, but become protonated in acidic endosomes, triggering membrane disruption and mRNA release [11,12]. Helper lipids such as dioleoylphosphatidylethanolamine (DOPE) enhance fusogenicity [13]; cholesterol provides structural rigidity and modulates phase transitions necessary for membrane fusion [14]; and PEG-conjugated lipids sterically stabilize particles, reduce protein adsorption, and prolong systemic half-life [15].

Despite this, the physicochemical and pharmacological constraints of LNPs remain significant. Following systemic administration, apolipoprotein E (ApoE) adsorption onto LNP surfaces mediates hepatocyte uptake via low-density lipoprotein receptors (LDLR), yielding a characteristic hepatic biodistribution [16,17]. While this intrinsic liver tropism is advantageous for hepatocyte-directed therapies, it severely limits extrahepatic targeting. Moreover, LNPs display sensitivity to pH fluctuations and temperature stress, complicating formulation stability and long-term storage [18]. Transient innate immune activation via complement activation or cytokine release has also been observed, constraining repeated dosing regimens [19].

These limitations have prompted a wave of molecular redesign within the LNP field. Fine-tuning of lipid architecture—through alterations in hydrophobic tail branching, linker degradability, and headgroup polarity—has been shown to modulate both ionization behavior and organ selectivity [20,21,22]. For example, increased tail branching has been associated with splenic and lymphatic delivery, whereas linear lipid structures favor hepatic localization [23]. Similarly, surface engineering approaches that conjugate peptides, antibodies, or saccharides enable receptor-mediated targeting of extrahepatic tissues [24,25]. While these advances have expanded LNP utility, they emphasize the growing need for alternative materials capable of delivering comparable efficiency with greater synthetic modularity and long-term stability [26].

Compared with LNPs, polymeric materials offer greater structural flexibility, potential for controlled or sustained release, and enhanced stability during storage. In contrast, LNPs generally provide higher transfection efficiency and have a well-established clinical performance record [1,6]. Cationic polymers such as PEI, PBAEs, and PAMAM dendrimers condense mRNA into nanoscale polyplexes, facilitating cellular uptake [5,11]. Their protonatable amines buffer endosomes, promoting cytosolic release [27]. However, excessive charge density, particularly in high-molecular-weight PEI, can compromise cell viability by destabilizing the membrane and inducing mitochondrial stress [28]. To mitigate these effects, biodegradable and stimuli-responsive polymer designs have emerged, including PBAEs with ester linkages that degrade under physiological conditions [29], PEGylated PEI variants that reduce surface charge and immunogenicity [28], and redox- or pH-sensitive polymers that release cargo in response to intracellular cues [30,31].

Hybrid lipid–polymer nanoparticles combine lipid fusogenicity with the structural integrity of polymeric cores, thereby improving both encapsulation efficiency and endosomal escape [32,33]. CARTs are dynamic carriers that initially bind mRNA via cationic charges but undergo intracellular structural rearrangement to neutral states, enabling efficient mRNA release with minimal toxicity. Similarly, poly(amino acid)s such as poly(L-ornithine), modified with pH-responsive charge-conversion or hydrophobic groups, are being engineered to enhance endosomal escape and reduce cytotoxicity.

The expanding therapeutic applications of mRNA require highly efficient mRNA delivery vectors. Advanced polymeric materials hold transformative potential in this context because their physicochemical properties can be precisely modified to optimize mRNA complexation, protect cargo from enzymatic degradation, and control intracellular release. Moreover, these polymers can be engineered to achieve organ- or cell-specific targeting, stimuli-responsive behavior, and compatibility with repeated dosing, addressing key limitations of conventional LNPs. By utilizing these unique advantages, polymeric systems are enabling the development of more effective, targeted, and personalized mRNA-based therapeutics.

## 3. Challenges in mRNA Delivery and Design Considerations

The therapeutic potential of mRNA is limited by substantial delivery challenges, due to its inherent chemical instability and the need to traverse various biological barriers [11]. Below, we outline key obstacles to clinical translation and discuss how current delivery platforms, including polymeric and hybrid systems, aim to address these limitations (Table 1).

### 3.1. mRNA Instability and Degradation

The single-stranded structure of mRNA renders it highly vulnerable to degradation by ubiquitous RNases, given that some unprotected mRNA displays a half-life of mere minutes in vivo [34]. Chemical modifications, such as N1-methylpseudouridine (m1Ψ), optimized 5′ caps, and poly-A tails, can enhance stability and translational efficiency but are insufficient alone to protect naked nucleic acids in biological environments [35]. In addition to their beneficial effects on mRNA stability, translational efficiency, and reduced innate immune activation, nucleoside modifications may also introduce safety-relevant considerations that require careful evaluation. For example, N1-methylpseudouridine (m1Ψ), which is widely used in clinically advanced mRNA vaccines, can reduce recognition by innate immune sensors such as RIG-I- and TLR-mediated pathways [36,37,38]. While this property can improve tolerability and protein expression, excessive reduction of innate immune activation may be undesirable in some therapeutic contexts, particularly where immune stimulation or tumor immune surveillance is relevant [36]. In addition, recent studies have shown that m1Ψ-containing mRNA can promote sequence-dependent +1 ribosomal frameshifting, leading to the production of unintended translation products [39]. These findings suggest that the use of modified mRNA requires careful sequence optimization, assessment of translation fidelity, and transparent reporting of RNA chemistry in preclinical and clinical mRNA delivery studies. The protection of mRNA during transfection, therefore, requires delivery vectors, such as LNPs and polymeric vectors. However, these systems must balance protection with payload release kinetics, particularly in applications requiring sustained protein expression [16].

### 3.2. Inefficient Cellular Uptake

The anionic charge of mRNA prevents passive diffusion across the cell membrane, necessitating carrier-mediated delivery. Cationic systems, which include PEI, PAMAM dendrimers, and lipid-polymer hybrids, neutralize mRNA’s charge to enable complexation and cellular internalization [5]. Insufficient surface charge, however, can lead to nonspecific interactions with serum proteins, reducing targeting precision [40]. To mitigate this, zwitterionic or other hydrophilic excipients are often incorporated to improve colloidal stability and minimize unintended protein adsorption. Nanoparticle size and surface chemistry further influence cellular uptake: particles larger than ~200 nm generally exhibit reduced internalization, and the effects of PEGylation depend on PEG chain length and cargo nature [35,41]. The development of pH-responsive poly(amino acid)s and charge-altering, releasable transporters thus optimizes charge dynamics and enhances uptake, while minimizing off-target interactions [42].

### 3.3. Endosomal Entrapment and Escape

Even when successfully internalized, mRNA often becomes trapped in endosomal compartments, where it is subject to degradative enzymes and an acidic environment [43]. For mRNA to exert its therapeutic effect, it must escape from endosomes into the cytoplasm, where translation can occur. PEI and PBAEs use buffering amines to disrupt endosomal membranes, although the escape efficiency varies widely [29,44]. Lipid-polymer hybrids and PBAEs enhance escape by combining controlled polymer degradation and membrane destabilization [1]. Although scalability, which is discussed in further detail as a separate issue, remains a challenge, emerging strategies—such as light- or redox-sensitive stimuli-responsive polymers—offer spatiotemporal control over mRNA release [45]. However, the efficiency of this process varies widely among platforms, and incomplete endosomal escape can result in mRNA degradation before it reaches the cytoplasm. Improving endosomal escape, therefore, remains a central objective in the design of delivery systems.

### 3.4. Immunogenicity and Toxicity

Pattern recognition receptors (PRRs) recognize unmodified mRNA in the immune system, triggering innate immune responses that may lead to inflammation and reduced therapeutic efficacy [19,46]. While chemical modifications help reduce immunogenicity, it is important to consider that mRNA instability or degradation may still generate fragments that activate PRRs [1]. Additionally, the cationic nature of many delivery systems can induce cytotoxicity and inflammatory responses, particularly at high doses [19]. PEI, a widely studied cationic polymer, is associated with significant cytotoxicity due to its high charge density and large size. The use of biocompatible polymers and low-molecular-weight derivatives, PEGylation to reduce surface charge, and addition of biodegradable linkages that minimize long-term toxicity are among the existing strategies to mitigate these effects [47,48].

### 3.5. Targeted Delivery and Organ Specificity

Targeted delivery and control of biodistribution further define the frontier of mRNA delivery science. The physicochemical parameters governing nanoparticle behavior in vivo—particle size, zeta potential, shape, and hydrophobicity—dictate organ tropism and cellular uptake [11,49,50]. Hepatic accumulation remains predominant due to the liver’s fenestrated sinusoidal endothelium and scavenger-rich microenvironment [51]. Strategies to achieve extrahepatic delivery include surface functionalization with targeting ligands (e.g., folate, galactose, mannose), modulation of PEG-chain length to influence protein-corona formation, and exploitation of administration routes favoring local uptake (such as intramuscular or pulmonary delivery) [52,53,54]. Yet true cell-type specificity—especially across complex tissues such as the brain or tumor microenvironment—remains elusive [55].

### 3.6. Scalability and Manufacturing Challenges

Additional challenges arise in the large-scale production of mRNA and its delivery systems. For regulatory approval and consistent biological performance, nanoparticles must be produced with consistent, well-defined size, composition, and encapsulation efficiency. LNPs, aided by mature microfluidic production platforms, can achieve control over mixing and self-assembly [32]. In contrast, polymeric nanoparticles and dendrimers often suffer from batch-to-batch variability as a consequence of polymer polydispersity or multi-step synthesis routes [56]. Simplified synthetic pathways, solvent-free processes, and continuous-flow microreactor systems are technologies being actively developed to address these limitations [57,58].

## 4. Recent Advances in Polymeric Vectors for mRNA Delivery

### 4.1. PEI

PEI remains the gold standard for DNA transfection, where its dense cationic charge facilitates strong condensation and nuclear transport [59,60,61]. However, these same interactions hinder mRNA release, as cytosolic translation requires rapid dissociation and reduced toxicity. DNA must traverse the nuclear envelope for transcription, whereas mRNA acts exclusively in the cytosol and is inherently more susceptible to enzymatic degradation [11]. Excessive electrostatic interactions may induce membrane destabilization, hemolysis, and cytotoxicity—especially pronounced in high-molecular-weight forms [62,63]. This has motivated a steady evolution of chemical modifications aimed at preserving its exceptional delivery capabilities while mitigating toxicity and, in some cases, imparting additional functionality.

One such approach, reported by Li et al., introduced fluoroalkane moieties onto a 1.8 kDa PEI backbone, generating a fluorinated derivative (F-PEI) with a distinctive combination of hydrophobic and lipophobic properties [64]. Notably, the modification endowed the polymer with intrinsic immunostimulatory capacity via Toll-like receptor 4 activation, enabling it to serve simultaneously as a carrier and adjuvant and to improve antitumor responses in vivo.

Where fluorination primarily reconfigures membrane interactions, other strategies have instead targeted colloidal stability and aggregation control. Tan et al. covalently linked β-cyclodextrin (β-CD) to branched PEI (2 kDa), creating CD-PEI conjugates in which the cyclodextrin cavity reduced particle aggregation and improved complex stability [65]. The PEI segment retained its mRNA condensation and endosomal escape capacity, but the overall construct exhibited lower cytotoxicity and higher transfection efficiency than unmodified PEI. Importantly, their in vivo studies revealed that immune responses varied significantly with the route of administration.

Hamada et al. addressed surface charge modulation by developing ternary complexes comprising PEI, γ-polyglutamic acid (γ-PGA), and mRNA [66]. The anionic γ-PGA shell masked the cationic charge of PEI, preventing erythrocyte aggregation and reducing nonspecific membrane interactions, without compromising delivery performance. These complexes preferentially accumulated in the liver and spleen in vivo, achieving high levels of protein expression—attributes well-suited to systemic and organ-targeted mRNA applications.

More recently, structural modification of the PEI backbone has been investigated to balance delivery efficiency with biocompatibility. Kim et al. reported kanamycin-polyethylenimine (KANPEI) conjugates in which low-molecular-weight PEI chains were grafted onto a degradable aminoglycoside scaffold, which enabled efficient mRNA condensation into nanoscale polyoplexes while maintaining strong buffering capacity for endosomal escape [67]. Transfection efficiencies comparable to those of PEI (25 kDa) were achieved with the optimized KANPEI 1.2 kDa formulation, but with markedly reduced cytotoxicity.

### 4.2. PBAE

Like PEI, PBAEs condense nucleic acids through electrostatic interactions; however, their hydrolytically degradable ester linkages confer superior biocompatibility. Synthesized via Michael addition between acrylates and amines, PBAEs enable precise control over molecular weight, charge density, and release kinetics [29]. Their tertiary amine-rich backbone facilitates a robust buffering capacity for endosomal disruption and cytosolic release, while ester linkages undergo gradual hydrolysis, mitigating long-term toxicity [68,69].

**Figure 2:**
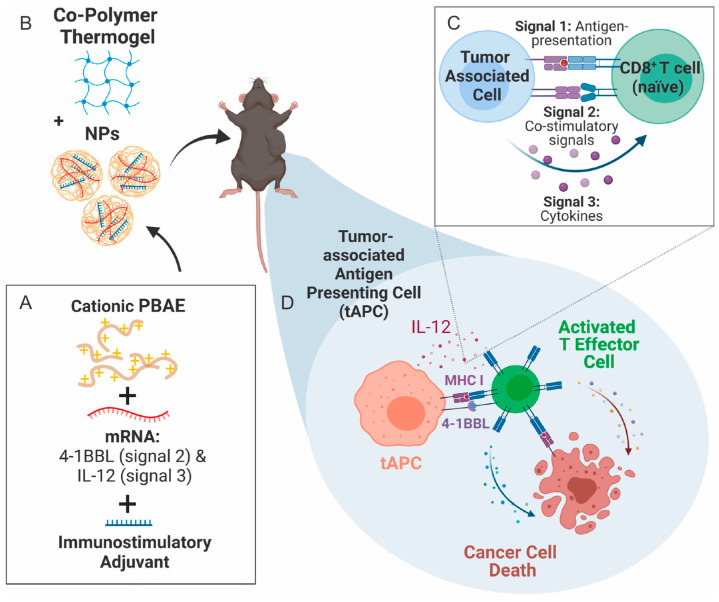
Schematic of mRNA nanoparticle (NP) fabrication and therapeutic mechanism. The fabrication process begins with (**A**) nanoparticle assembly, where mRNA encoding therapeutic signals 2 and 3 is complexed with cationic poly(beta-amino ester)s (PBAEs) and an immunostimulatory adjuvant. These NPs are subsequently (**B**) formulated with thermoresponsive PLGA-PEG-PLGA polymers for intratumoral injection. Upon delivery, the (**C**) NPs transfect tumor-associated cells, programming them to express signal 2 and secrete signal 3. This transformation allows them to function as (**D**) tumor-associated antigen-presenting cells (tAPCs), which effectively engage and activate T effector cells to coordinate a targeted attack against cancer cells. Reprinted with permission from ref. [68]. Copyright 2023, Elsevier.

In certain studies, PBAEs have been harnessed to deliver mRNA encoding immunostimulatory cytokines and checkpoint modulators directly to the tumor microenvironment. An example is provided by Neshat and colleagues, in their design of PBAE-based nanoparticles to co-deliver mRNA encoding 4-1BB ligand, a T-cell costimulatory molecule, and IL-12, a pro-inflammatory cytokine, which synergistically enhanced T-cell activation and antitumor immunity in murine breast and colorectal cancer models (Figure 2) [68]. This approach, combined with anti-PD1 checkpoint blockade, achieved complete tumor regression in several cases.

Beyond parenteral administration, PBAEs are also being explored for non-invasive mRNA delivery. Kim et al. developed a branched hybrid PBAE (bhPBAE) polymer for oral mRNA vaccines with the aim of targeting immune-rich regions in the gastrointestinal tract, such as Peyer’s patches [69]. The bhPBAE nanoparticles delivered mRNA encoding model antigens such as ovalbumin efficiently, eliciting robust systemic and mucosal immune responses while reducing cold-chain dependence.

For systemic and organ-selective delivery, Kavanagh et al. reported ligand-free PBAEs capable of achieving preferential pulmonary mRNA expression following intravenous administration, demonstrating the ability to tune tropism via polymer architecture alone [70]. Similarly, Hu et al. showed that the supramolecular assembly state of PBAE/mRNA nanoparticles dictates biodistribution and immune engagement, where small, weakly protonated assemblies preferentially engage circulating monocytes, thereby modulating downstream cytokine responses [71]. These findings highlight nanoassembly dynamics, rather than exogenous targeting ligands, as key determinants of tropism.

Efforts to further refine PBAE performance include combinatorial synthesis and high-throughput screening to systematically examine how structural variation influences delivery efficiency. Tu et al. developed a diverse library of PBAE derivatives bearing hydrophobic, aromatic, and hydrogen-bonding side chains to identify carriers capable of efficient mRNA delivery while exhibiting intrinsic adjuvant activity [72]. Lead formulations activated dendritic cells through Toll-like receptor signaling and, when loaded with ovalbumin or glycoprotein 100 mRNA, induced strong CD8^+^ T-cell responses, increased IFN-γ and TNF-α secretion, and significantly suppressed tumor growth in vivo. Kim et al., on the other hand, expanded the PBAE monomer repertoire to fine-tune cationic density and hydrophobic balance, yielding nanoparticles with high mRNA encapsulation, prolonged expression lasting up to two weeks, and minimal hepatic toxicity [73].

### 4.3. Dendrimers

Dendrimers are highly branched, monodisperse synthetic macromolecules with a well-defined tree-like architecture, which enables precise control over size—typically 1 to 10 nm—as well as surface functionality and molecular weight [74]. With a structure consisting of a central core, repeated branching units, and numerous terminal functional groups, dendrimers have high versatility in surface modification and cargo loading [75]. These features allow them to efficiently interact with cellular membranes, supporting their use in nucleic acid delivery [76].

Khazaei Monfared and co-workers leveraged dendrimer functionalization in the application of dendrimers in spatially controlled therapies [77]. They designed a cationic hyper-branched cyclodextrin-based polymer for the intratumoral delivery of mRNA, achieving efficient transfection and robust antigen-specific CD8^+^ T-cell activation, with superior stability compared with Lipofectamine and free mRNA. Effective mRNA expression in human nucleus pulposus and fibroblast-like synoviocytes using poly(amidoamine)-based nanoparticles was demonstrated by Muenzebrock et al. [78]. The use of polyglutamic acid-polyethylene glycol (PGA-PEG) surface coatings enhanced nanoparticle stability and reduced cytotoxicity, highlighting the promise of surface engineering for cellular compatibility.

Beyond surface modification, recent work has explored stimuli-responsive dendrimer architectures for temporally controlled cargo release. Li et al. reported self-immolative Janus dendrimers composed of hydrophilic oligo(ethylene glycol) dendrons and hydrophobic oligo(ethyl glyoxylate) chains that self-assemble into spherical nanoparticles [79]. UV-induced cleavage of a photoresponsive endcap triggered rapid depolymerization of the hydrophobic segments, prompting a morphological transition from spherical to crescent-shaped particles and allowing controlled release of encapsulated cargo.

Systemic delivery has similarly benefited from architectural optimization [80,81]. Hardenberg et al. demonstrated effective encapsulation of hemagglutinin mRNA by poly(amidoamine)-based nanoparticles in influenza models, inducing strong humoral and cellular immune responses [80]. Pontes et al. further improved intracellular delivery by incorporating chloroquinoline (Q) moieties into a poly(amidoamine) (PAA)-based polymeric nanoparticle (PNP) platform [81]. Additional PEG coating of ps-PAAQ nanoparticles loaded with luciferase mRNA exhibited significantly higher transfection efficiency in vivo compared to uncoated formulations. Complementary work has highlighted the importance of pH-responsive tertiary amine functionalities for intracellular delivery. Zhao et al. used biodegradable tertiary amine polymers derived from a PAMAM dendrimer backbone in lipid nanoparticle formulations [82]. The optimized formulation achieved ~80% endosomal escape (compared with ~20% for standard LNPs) and produced up to a 100-fold increase in hepatic luciferase expression in vivo while maintaining high mRNA encapsulation efficiency (>90%) and minimal inflammatory cytokine release.

### 4.4. Polyesters

Unlike cationic polymers, which rely on electrostatic interactions for nucleic acid complexation, classical polyesters such as poly(lactic-co-glycolic acid) (PLGA) and poly(amine-co-ester) (PACE) leverage their chemically adaptable backbones, which can be engineered with cationic or hydrophobic moieties, to achieve multifunctionality [83]. This improves mRNA binding, nanoparticle stability, and intracellular trafficking, while maintaining their inherent biodegradability, allowing for safe metabolic clearance—a critical feature for minimizing long-term toxicity [83,84]. Polyesters are also compatible with scalable manufacturing techniques, positioning them as a clinically viable platform for sustained and targeted mRNA therapies.

PACE polymers exemplify this approach. Synthesized via condensation polymerization of lactone monomers and amino diols, they form stable polyplexes capable of efficiently delivering mRNA to targeted tissues when modified with specific end groups [1]. Suberi et al. demonstrated efficient lung-localized expression and robust immune responses following SARS-CoV-2 mRNA vaccination using PACE polyplexes, with minimal inflammation [85]. The “4Q” principle was proposed to guide the design of cationic polycatechols, such as poly(N,N’-bis(acryloyl)-cystamine-co-dopamine) (PBD), which exhibited >2-week stability at room temperature and higher in vivo transfection efficiency compared to jetPEI [86]. By considering multiple delivery factors, this approach provided a relevant framework for the optimization of polymeric vectors for clinical translation.

Efforts to mitigate inflammatory responses have also driven progress in polyester design. A series of ortho-hydroxy tertiary amine (HTA) copolymers (PHTA) developed by Huang et al. to form stable polymeric nanoparticles (PNPs) showed minimal inflammatory responses, with its top candidate (PHTA-C18) demonstrating significant antitumor efficacy in a melanoma model [87].

### 4.5. CARTs

CARTs form a distinct class of mRNA delivery vectors that use a pH-sensitive degradation mechanism [88,89]. Upon exposure to physiological conditions, the polycationic segment undergoes an oxygen-to-nitrogen (O-to-N) acyl shift, resulting in the synthesis of neutral lactams.

**Figure 3:**
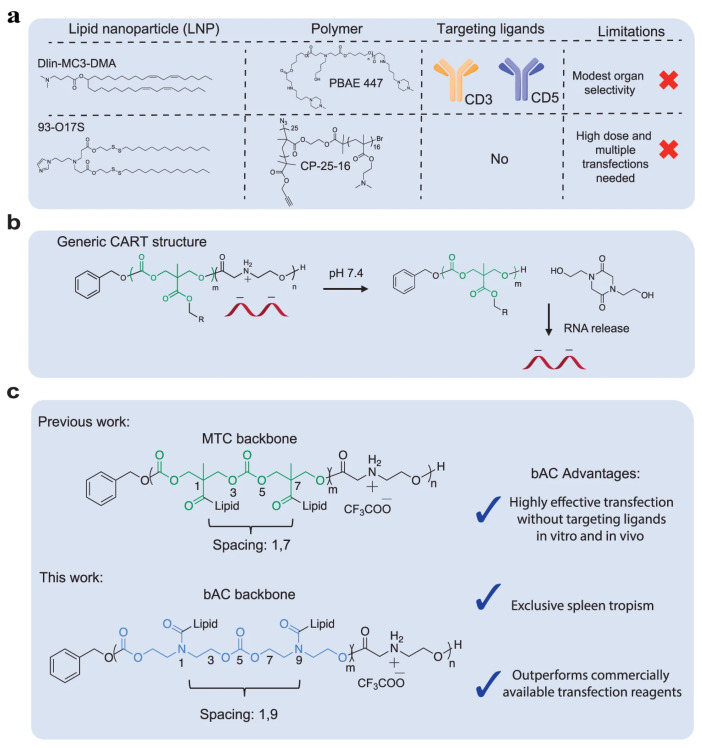
Architecture and release mechanism of the CART delivery system. This figure illustrates (**a**) a representative nonviral platform specifically engineered for primary T-cell transfection. Central to this system are (**b**) CART/mRNA complexes, which utilize a pH-driven nitrogen-to-oxygen acyl shift to trigger charge cancellation, thereby facilitating controlled mRNA release. Furthermore, (**c**) bAC CARTs are shown to possess a specialized polymeric backbone with optimized lipid spacing, a structural refinement that significantly enhances delivery efficiency to T cells. Reprinted with permission from ref. [90], licensed under CC BY 4.0. Copyright 2023, the Authors.

Li et al. introduced a series of beta-amido carbonate (bAC) CARTs, in which backbone modifications and side-chain spacing were adjusted to influence hydrophobicity, nanoparticle stability, and release kinetics, as seen in Figure 3 [90]. These materials achieved up to 70% transfection of primary T lymphocytes and 97% spleen tropism in vivo, without the need for targeting ligands or detectable inflammatory responses.

In a related advance, the same group developed guanidinylated serinol (GSer) CARTs, where guanidinium groups strengthened electrostatic and hydrogen-bond interactions with mRNA but underwent guanidine-to-carbonate cyclization at physiological pH to ensure release [91]. By systematically varying lipid chemistry, polymer length, and the guanidinium-to-phosphate charge ratio, the authors demonstrated precise control over organ tropism—achieving up to 96% lung or 98% spleen selectivity after intravenous administration.

Structural tuning of the cationic block can also alter biodistribution. Blake et al. replaced glycine-derived segments with lysine-based cations, which shifted tropism from the spleen to the lungs [92]. Rahn and co-workers explored lipid architecture using isoprenoid-CARTs functionalized with archaeal-inspired branched lipids [88]. Shorter branched tails, such as isoamyl, achieved high transfection in lymphoblastic cell lines, whereas longer chains favored epithelial cell targeting.

### 4.6. Poly(amino acid)s

As they are typically synthesized via ring-opening polymerization of amino acid-based monomers, poly(amino acid)s allow for the incorporation of functional groups that can enhance stability, cellular uptake, and endosomal escape of mRNA cargoes [2]. The functional side chains attached to poly(amino acid) backbones—such as amines, carboxylates, or hydrophobic residues—can be tailored to enhance various considerations, such as mRNA complexation and nanoparticle stabilization [93]. For example, Kim et al. investigated how side-chain chemistry influences delivery performance using a series of amphiphilic polyaspartamide [PAsp(R/CHE)] derivatives bearing different amine functionalities [94]. Polymers containing diethylenetriamine (DET)-based moieties showed the highest mRNA expression, which can be attributed to the increased buffering capacity of DET and enhanced endosomal escape.

Park et al. similarly used DET-substituted poly(aspartic acid) [P(Asp(DET))]-based polymeric nanoparticle (PNP) to explore both local and systemic mRNA delivery [95]. Control of biodistribution and transfection efficiency was possible by varying the density of PEGylation of P(Asp(DET)). Moderate PEGylation (1:1) promoted lung-targeted delivery following intravenous injection, whereas higher PEG content (10:1) favored protein expression following intramuscular injection, highlighting the role of PEG density in route-specific mRNA delivery.

Dirisala and colleagues investigated poly(L-ornithine) in combination with a charge-conversion polymer for mRNA delivery [44]. The coupling of poly(L-ornithine) ‘s strong mRNA-binding with endosomal destabilization enabled efficient endosomal escape under acidic conditions, allowing for controlled mRNA release into the cytoplasm. These findings suggest a promising direction for mRNA delivery in sensitive or hard-to-transfect cell populations.

### 4.7. Natural Cationic Polymers

Natural cationic polymers, particularly polysaccharide-based systems such as chitosan and cationic derivatives of dextran and hyaluronic acid, represent biologically compatible alternatives to fully synthetic gene delivery vectors. Among these, chitosan—a partially deacetylated derivative of chitin composed of β-1,4-linked glucosamine units—has been most extensively investigated [96]. Its protonatable amine groups allow electrostatic complexation with mRNA, forming polyplexes which protect mRNA cargo and support cellular uptake.

Recent work by Kota et al. explored chitosan-tripolyphosphate (CS–TPP) nanoparticles for inhalable mRNA delivery. The formulations demonstrated successful aerosolization, efficient cellular uptake in A549 and BEAS-2B cell lines, and measurable protein expression following transfection [97]. Their work highlighted the feasibility of pulmonary delivery, with aerodynamic particle sizes optimised (1–5 µm) for deep lung deposition.

However, native chitosan systems remain limited by relatively low transfection efficiency. Mechanistically, this is largely due to poor solubility at physiological pH and insufficient buffering capacity, which raises the relevance of chemical modification and hybridization strategies [98]. In this regard, Xu and colleagues engineered an amphiphilic chitosan-PEI hybrid (PAN2H), in which palmitic acid-modified quaternised chitosan was combined with PEI-mRNA complexes [99]. This system achieved approximately twofold higher mRNA expression in vitro compared to PEI alone, alongside enhanced in vivo immunogenicity, illustrating how amphiphilicity and combined electrostatic interactions can improve delivery performance. More broadly, modifications such as quaternization and carboxymethylation have been used to improve chitosan solubility and gene release efficiency by altering charge density and polymer-RNA interactions [98].

### 4.8. Hybrid Polymeric Vectors

**Figure 4:**
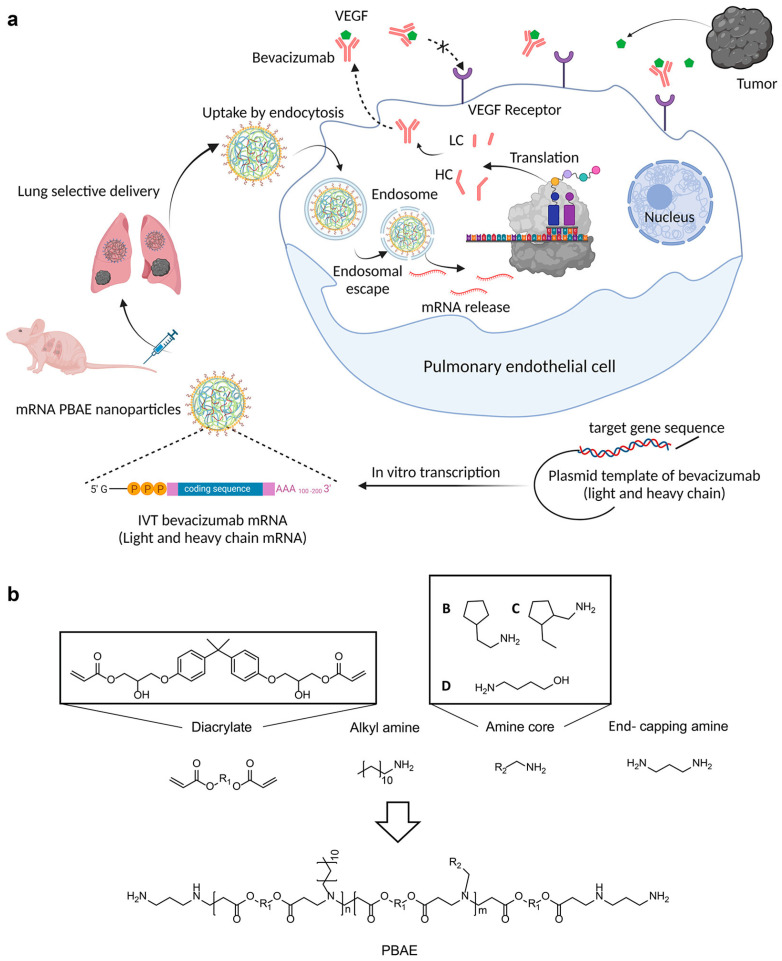
Mechanism and library of the PBAE-delivered bevacizumab mRNA therapy. This figure presents (**a**) a schematic illustration of the hypothetical mechanism of action for bevacizumab mRNA therapy in treating non-small cell lung cancers (NSCLCs). Encapsulated within PBAE nanoparticles (NPs), the mRNA transcripts for bevacizumab’s heavy and light chains are administered intravenously, selectively distributing to pulmonary endothelial cells (PECs). Subsequent transfection results in the local secretion of bevacizumab, which neutralizes tumor-derived VEGF to inhibit angiogenesis. Additionally, the figure displays (**b**) the library of PBAE polymers synthesized and evaluated within this study. Reprinted with permission from ref. [100]. Copyright 2024, American Chemical Society.

By combining the biocompatibility and fusogenic properties of lipids with the structural stability and adjustable charge density of polymers, lipid-polymer hybrid systems address critical challenges in mRNA therapeutics, including stability, targeted delivery, and endosomal escape. Recent studies illustrate the potential of these systems across diverse applications, from cancer immunotherapy to vaccine development. For example, PBAEs, which were combined with lipids such as DMG-PEG and DOPE by Le et al. [100]. Increasing amino groups in PBAE-D polymers were shown to improve mRNA complexation, while optimizing PEG lipid tail length and molarity refined biodistribution. Their DSPE-PEG-PBAE formulation demonstrated therapeutic efficacy in non-small cell lung cancer (NSCLC) models and successfully delivered bevacizumab mRNA to block VEGF in pulmonary tissues. The proposed mechanism of lung-selective delivery and mRNA translation is illustrated in Figure 4. Similarly, Cao et al. [101] developed five-element nanoparticles (FNPs) integrating PBAEs with DOTAP, DOPE, cholesterol, and DMG-PEG; FNPs incorporated with longer PBAE side chains and optimal DOTAP ratios were most successful in achieving mRNA delivery efficiency and exhibited excellent in vivo tolerance and organ-specific accumulation.

Lipid-polymer hybrids have furthermore shown exceptional promise in oncology. Chen and co-workers reported vesicle-like nanoparticles (VNPs) composed of phospholipids, cholesterol, and PEG derivatives, achieving >90% mRNA encapsulation and 60–70% transfection efficiency in cancer cells (HeLa, MCF-7), with sustained stability, strong tumor accumulation, and resultant reduction of tumor volume by over 50% [102]. Kliesch et al. designed PLGA core lipid-shell nanoparticles (DOTMA/DOPE) that improved endosomal escape and cellular uptake, although higher DOPE ratios increased free mRNA, suggesting that a balance is critical [33].

The administration route also critically influences performance. Meyer et al. showed that PLGA-based hybrids had superior lung and spleen expression when mRNA was surface-adsorbed, whereas encapsulated formulations excelled in intramuscular delivery, retaining activity for 12 days [103]. Findings by Hendy et al., in which COBRA mRNA LNPs elicited robust neutralizing antibodies against influenza but showed reduced immunogenicity when combined with cGAMP microparticles, emphasize the importance of formulation optimization [104].

Lipidation strategies have also been applied to cationic polymers to further refine hybrid architectures. In one example, Chen and colleagues modified PEI with long C14 alkyl chains and then coated it with anionic PEG–poly(L-glutamic acid). This two-step modification produced nanoparticles with a near-neutral surface charge and improved stability under physiological conditions, delivering mRNA efficiently and triggering fewer inflammatory cytokine responses in vivo, suggesting broad therapeutic utility. Similarly, Wang et al. described PEI-based lipopolymers (ALL-Fect, Leu-Fect C) with similarly high transfection efficiency and minimal toxicity, although the mechanistic basis for their spleen/liver tropism remains unclear [105]. Ben-Akiva et al. focused on dendritic cell-targeted formulations of lipid-modified PBAEs, which enhanced antitumor immunity through co-delivery of TLR agonists (CpG, poly(I:C)) and mRNA [106].

### 4.9. Other Polymeric Vectors

Beyond the established polymer classes described above, there is a need to highlight several innovative systems developed recently. For instance, Rodrigues and co-workers employed a high-throughput combinatorial approach to synthesize 152 chemically diverse polycation and identified a lead formulation with pronounced fibroblast tropism. This nanoparticle efficiently transfected skin fibroblasts much more efficiently than other co-resident cell types and even outperformed conventional lipid-based vectors such as Lipofectamine, for delivering mRNA, including Cas9 mRNA with sgRNA. Fibroblast-selective uptake occurred via receptor-mediated endocytosis involving cell-surface markers CD26 and FAP, suggesting that intrinsic polymer chemistry without appended ligands can drive cell specificity. Structure–activity analysis of the polymer library further indicated that efficient mRNA transfection required a combination of high buffering capacity and low mRNA-binding affinity [107]. Such findings underscore the power of combinatorial polymer design to pinpoint chemical features for selective mRNA delivery in specialized disease contexts, such as for fibrotic tissues.

Another emerging strategy is the use of stimuli-responsive polymeric nanostructures to improve in vivo stability. Yang et al. reported a block copolymer, PEG-PLL(CAA), consisting of poly(ethylene glycol) coupled to poly(l-lysine) chains partially modified with cis-aconitic anhydride (CAA) [108]. This amphiphilic polymer forms polyion complex (PIC) micelles with mRNA, which are highly stable under physiological conditions (pH 7.4) and capable of resisting premature dissociation or polyanion exchange, but dissolve in the acidic endosomal environment (pH ~5.5) to release the mRNA cargo. The formulation showed improved protein expression compared with non-crosslinked controls, highlighting the benefit of temporary core stabilization. PEGylation simultaneously improved circulation time and reduced immunogenicity, while allowing room for further optimization, such as tuning the PEG chain length or introducing targeting ligands.

Targeted glycopolymer vectors extend this concept to specific immune cell populations. Fan et al. synthesized a dendritic-cell-targeted polymeric nanoparticle system, featuring an aryl trimannoside ligand to engage DC-SIGN receptor, combined with guanidinium-rich backbones for mRNA complexation and disulfide linkages for reductive intracellular cleavage [109]. This sophisticated glyco-functionalized polycation was effective at potently transfecting mRNA into dendritic cells in vitro and in vivo, resulting in an enhanced immune response against the encoded SARS-CoV-2 spike protein and, most notably, outperforming untargeted polymer analogs.

## 5. Discussion

The landscape of mRNA therapeutics is advancing rapidly, and polymeric carriers are playing an increasingly important role in addressing persistent challenges, including instability, unwanted immunogenicity, and inefficient cellular uptake. Compared to their viral-vectored counterparts, mRNA offers advantages in speed of production and the capacity to elicit potent immune responses, as demonstrated during the development of SARS-CoV-2 vaccines [110]. LNPs have been central to early clinical translation, yet limitations in tissue selectivity, biodistribution, storage stability, and systemic tolerability have driven the search for alternative platforms. Polymers, with their broad synthetic tunability, enable precise control over nanoparticle parameters—including size, charge, degradability, and targeting—thereby positioning them as adaptable, highly customizable systems for next-generation mRNA delivery. Over the past five years, research on polymeric vectors for mRNA delivery has evolved from proof-of-concept polyplex formation and in vitro transfection toward rationally engineered, biodegradable, organ-selective, and cell-targeted nanocarriers. The field has moved through several stages: first responding to the limitations of LNP-dominated mRNA delivery, then developing chemically diverse polymer architectures, followed by high-throughput screening for tissue tropism, structure–activity-guided optimization of PBAE/HPAE and CART systems, and most recently, translational applications such as inhaled lung delivery, mRNA cancer vaccination, and in vivo immune-cell engineering. This evolution suggests that polymeric vectors are emerging not merely as backup systems to LNPs, but as a distinct and highly tunable platform for next-generation mRNA therapeutics. Although most of the polymeric mRNA delivery techniques are still in R&D and preclinical studies, early clinical translation of the hybrid system is now underway. A phase II trial (NCT07321301) is currently recruiting participants to evaluate polymer-lipid nanoparticle delivery of CD19/CD20 dual-targeting mRNA in CAR-T cell therapy for relapsed/refractory B-cell lymphoma and leukemia—representing a first-in-class demonstration of the combinatorial potential of polymeric and lipid-based platforms.

Across the platforms reviewed here—including PEI derivatives, PBAEs, poly(amino acid)s, polyesters, dendrimers, charge-altering releasable transporters (CARTs), and lipid-polymer hybrids—no single material class is universally optimal. Instead, performance depends on matching the carrier design to the therapeutic goal, administration route, and target tissue. For example, biodegradable backbones and stimuli-responsive linkages can improve tolerability and promote cytosolic release, while ligand display or chemistry-driven tropism can enhance cell selectivity.

Despite these advances, translational challenges nevertheless persist. Significant barriers include variability in polymer synthesis, batch-to-batch reproducibility, and the need for GMP-compliant manufacturing for both polymers and formulated nanoparticles. In parallel, comprehensive in vivo studies are needed to understand innate immune activation, complement responses, and clearance of polymer degradation products, especially for systemic and repeat-dose regimens.

The interaction between RNA chemistry and delivery vehicle design is an important but still underexplored consideration in mRNA therapeutics. At present, polymeric vectors should not be claimed to eliminate the need for modified mRNA; rather, their main potential may lie in reducing the required RNA dose and enabling systematic investigation of how carrier chemistry and RNA base modification jointly determines expression, safety, and therapeutic performance. In principle, highly efficient polymeric vectors may reduce the amount of mRNA required to achieve a therapeutically relevant level of protein expression, thereby indirectly reducing the total exposure to modified mRNA. However, current evidence remains insufficient to conclude that polymeric systems can generally replace the need for modified nucleosides or consistently reduce modRNA-associated biological effects compared with LNPs. Available studies show that the influence of modified nucleosides on protein expression is highly context-dependent and varies with mRNA sequence, modification type, cell type, administration route, and delivery vehicle. In some systems, modified mRNA enhances expression and reduces innate immune activation, whereas in others, unmodified or less-modified RNA may achieve comparable or even superior expression, particularly when paired with optimized carriers. Therefore, the relationship between polymer chemistry and RNA base modification should be considered a key design variable rather than a fixed requirement. Future studies should systematically compare unmodified, partially modified, and fully modified mRNA within the same polymeric delivery platform and benchmark these formulations against LNPs in terms of expression efficiency, innate immune activation, cytotoxicity, translation fidelity, and therapeutic index.

Looking ahead, high-throughput polymer synthesis and screening, combined with data-driven modeling (including machine learning), are likely to accelerate the discovery of carriers with improved efficacy and selectivity. Rational integration of polymers with lipids or other functional components, and co-delivery of mRNA with adjuvants or gene-editing machinery, should further expand the clinical reach of mRNA medicines.

From a critical perspective, the promise of polymeric mRNA delivery should be interpreted with caution. Many reported systems remain at the proof-of-concept stage and are evaluated mainly by short-term reporter-gene expression, with limited evidence of therapeutic efficacy, repeat-dose safety, or scalable manufacturing. In addition, claims of superiority over LNPs are often based on indirect comparisons rather than standardized head-to-head studies. The same chemical tunability that makes polymers attractive also increases the complexity of synthesis, characterization, reproducibility, and regulatory control. Therefore, future work should focus less on generating additional polymer variants and more on establishing robust design rules, standardized benchmarking against LNPs, clinically relevant disease models, and GMP-compatible manufacturing strategies. In our view, polymeric vectors should not be considered universal replacements for LNPs, but rather complementary and application-specific platforms whose value will depend on whether they can solve delivery problems that lipid-based systems cannot adequately address.

Overall, continued interdisciplinary development spanning polymer chemistry, formulation science, immunology, and manufacturing will be essential to translate polymer-enabled mRNA delivery from promising preclinical systems into robust, widely deployable therapies.

## Figures and Tables

**Table 1:** Key delivery barriers for mRNA therapeutics and representative polymer design strategies.

Barrier	Underlying Issue	Design Strategies Commonly Used in Polymeric Carriers
Instability and RNase degradation	Rapid hydrolysis/enzymatic cleavage of unprotected mRNA	Condensation/encapsulation; steric shielding; stabilizing interactions; optimized release kinetics
Cellular uptake	Anionic cargo and limited membrane permeability	Charge tuning; size control (<~200 nm); surface chemistry to manage protein corona; receptor targeting
Endosomal escape	Entrapment and degradation in the endolysosomal pathway	Buffering capacity/proton sponge; membrane-active motifs; degradable or pH-responsive linkers; hybrid lipid components
Immunogenicity and toxicity	PRR activation; cationic membrane disruption	Biodegradable backbones; lower MW; shielding (PEG/zwitterions); controlled release; dose/route optimization
Organ/cell specificity	Liver-dominant accumulation after systemic dosing	Ligand display; physicochemical tropism; route optimization; hybrid systems; local delivery depots
Manufacturing and scalability	Polymer polydispersity; complex synthesis; formulation reproducibility	Robust synthetic routes; defined feed ratios; scalable mixing; rigorous QC; GMP-compatible processes

## Data Availability

No new data were created or analyzed in this study. Data sharing is not applicable to this article.
